# The Dynamics of the Neutrophil-to-Lymphocyte and Platelet-to-Lymphocyte Ratios Predict Progression to Septic Shock and Death in Patients with Prolonged Intensive Care Unit Stay

**DOI:** 10.3390/medicina59010032

**Published:** 2022-12-23

**Authors:** Ioana Denisa Botoș, Carmen Pantiș, Constantin Bodolea, Andrada Nemes, Dana Crișan, Lucreția Avram, Marcel Ovidiu Negrău, Ioana Elisabeta Hirișcău, Rareș Crăciun, Cosmin Ioan Puia

**Affiliations:** 1 Faculty of Medicine and Pharmacy, University of Oradea, 410068 Oradea, Romania; 2 Intensive Care Unit, Clinical Municipal Hospital, 400139 Cluj-Napoca, Romania; 3 Faculty of Medicine, “Iuliu Hațieganu” University of Medicine and Pharmacy, 400012 Cluj-Napoca, Romania; 4 Department of Internal Medicine, Clinical Municipal Hospital, 400139 Cluj-Napoca, Romania; 5 Gastroenterology Clinic, “Prof. Dr. O. Fodor” Regional Institute of Gastroenterology and Hepatology, 400162 Cluj-Napoca, Romania; 6 Department of Surgery, “Prof. Dr. O. Fodor” Regional Institute of Gastroenterology and Hepatology, 400162 Cluj-Napoca, Romania

**Keywords:** sepsis, septic shock, intensive care, neutrophil-to-lymphocyte ratio, platelet-to-lymphocyte ratio, hospital-acquired infections

## Abstract

*Background and objectives:* The prognoses of patients experiencing a prolonged stay in the intensive care unit (ICU) are often significantly altered by hospital-acquired infections (HAIs), the early detection of which might be cumbersome. The aim of this study was to investigate the roles of the neutrophil-to-lymphocyte (NLR), derived-NRL (d-NLR), platelet-to-lymphocyte (PLR), and lymphocyte-to-C-reactive protein (LCR) ratios in predicting the progression to septic shock and death. *Materials and Methods*: A retrospective analysis of a consecutive series of ninety COVID-19 patients with prolonged hospitalization (exceeding 15 days) admitted to the ICU was conducted. The prevalence of culture-proven HAIs throughout their hospital stays was documented. NLR, dNLR, PLR, and LCR were recorded on admission, day 7, and day 14 to assess their discriminative prowess for detecting further progression to septic shock or death. *Results:* The prevalence of HAIs was 76.6%, 50% of patients met the criteria for septic shock, and 50% died. The median time to the first positive culture was 13.5 days and 20.5 days for developing septic shock. Mechanical ventilation was a key contributing factor to HAI, septic shock, and mortality. On admission and day 7 NLR, dNLR, PLR, and LCR values had no prognostic relevance for events occurring late during hospitalization. However, day-14 NLR, dNLR, and PLR were independent predictors for progression to septic shock and mortality and have shown good discriminative capabilities. The AUCs for septic shock were 0.762, 0.764, and 0.716, while the values for predicting in-hospital death were 0.782, 0.778, and 0.758, respectively. *Conclusions:* NLR, dNLR, and PLR are quick, easy-to-use, cheap, effective biomarkers for the detection of a more severe disease course, of the late development of HAIs, and of the risk of death in critically ill patients requiring a prolonged ICU stay.

## 1. Introduction

Nearly three years have passed since the emergence of the COVID-19 pandemic, in December 2019, with a related death toll exceeding 6.5 million, as reported by the World Health Organization (https://covid19.who.int, accessed on 26 September 2022), and numerous other significant consequences expanding well beyond the realm of healthcare and medicine. However, there appears to be room for optimism, as the rate of severe disease forms and COVID-19-associated mortality is on a steady decline, generated by vaccination, widespread exposure to the infection, alterations in viral variants, and improved medical care in severe and critical forms [1,2,3]. Given the novelty of the SARS-CoV-2 infection and the global impact of the pandemic, there has been a widespread tendency to intensify patient enrollment in research scenarios, aiming to extract any valuable data which could help the collective efforts. While most of these published data have helped to guide and improve patient care in the acute COVID-19 infection waves, there appear to be meaningful messages that might be extrapolated in multiple other clinical situations.

One key prognostic determinant in patients with COVID-19 has been acquiring a secondary co-infection during their hospital stay, impacting the entire disease severity spectrum [4,5,6]. Yet, the most vulnerable population to co-infections has consisted of patients with severe COVID-19 requiring admission to an intensive care unit (ICU) [5,7]. Cumulative factors determine the vulnerability of critically ill patients to secondary bacterial and fungal infections. These include organ failures, immune and metabolic dysfunction, the need for invasive procedures (mechanical ventilation), and, not least, a higher propensity for multi-drug resistant infections in the ICUs [7,8,9]. The vast majority of secondary infections in the ICU are hospital-acquired infections (HAIs). Often mislabeled as a poor performance metric, discussing and reporting on the incidence, outcomes, and antimicrobial management of HAIs is disproportionately less frequent compared to other variables, the topic often being treated as the elephant in the room [10]. There is an epistemological argument that a higher prevalence of HAIs might be linked to the complexity of the cases treated, hospital type, and patient flow. Consequently, this generates a U-shaped prevalence curve with the two peaks represented on the one hand by the low-performance centers and on the other hand by the high-volume, high-performance tertiary care facilities.

Given the prognostic impact of secondary infections in the critically ill, the critical step in patient management appears to be identifying the first hints of infection using cheap, quick, and easy-to-use tools. Among the biomarkers investigated for this purpose have been cell-count ratios: neutrophil-to-lymphocyte (NLR), derived-NLR (d-NLR), platelet-to-lymphocyte (PLR), or lymphocyte-to-C-reactive protein (LCR). Two large-scale meta-analyses suggest significant associations between NLR, among other ratios, and various outcomes, including disease severity, length of hospital stay, and mortality [11,12]. However, most of the studies included in these meta-analyses include admission or peak ratios. Therefore, these data highlight the prognostic value of these biomarkers grossly, without allowing extrapolation to specific subgroup analyses. A more thorough review of the available literature reveals that using the peak values rather than the on-admission ratio might reveal more actionable information [13], yet little is known about the sensitivity of these biomarkers for the detection of complications such as HAIs.

The current study aimed to evaluate the role of four easy-to-use biomarkers, namely NLR, dNLR, PLR, and LCR, repeated weekly, in predicting the further progression towards late septic shock and mortality in critically ill patients with COVID-19 and experiencing prolonged hospitalization requiring admission to an ICU. As secondary objectives, our study aimed to describe the burden of HAIs in a tertiary-care ICU dedicated to managing COVID-19.

## 2. Materials and Methods

### 2.1. Research Structure and Study Population

This was a retrospective, observational, and longitudinal study. Data were collected in a single center, the ICU of a tertiary-care hospital temporarily transformed into a high-volume dedicated COVID-19 facility. The patients were enrolled during a four-month timeframe, spanning from December 2020 to March 2021. Data on the same cohort of patients were previously reported in a study published by our team regarding the role of nutritional risk assessment tools in predicting in-hospital mortality [14]. All patients underwent a positive real-time reverse-transcriptase polymerase chain reaction (rRT-PCR) test for SARS-CoV2 within the first 24 h of their hospital stay and required ICU admission for COVID-19-associated complications. The inclusion criteria included the requirement of a prolonged hospitalization (exceeding 15 days). Patients with incidental findings of SARS-CoV-2 infection but admitted to the ICU for other complications (unrelated to the infection), a group that comprised patients with trauma, patients that required emergency surgery, or patients with other medical conditions with no or only mild lung involvement, were not included in the study.

### 2.2. Baseline Evaluation, Laboratory Workup, and Therapeutic Management

Patient history and demographic data were recorded on arrival. We standardized the comorbidity burden using the Charlson Comorbidity Index [15]. A comprehensive laboratory setup was performed and recorded within the first day of hospitalization, including complete blood counts, markers of inflammation (C-reactive protein—CRP, procalcitonin), coagulation, kidney, liver function, electrolytes, and metabolic balance. Interleukin-6 (IL-6) was recorded at the time of ICU admission. Per hospital protocol, patients were re-evaluated on demand at the clinician’s discretion and systematically on days 7 and 14. The Total Severity Score (TSS), computed on a standard thoracic CT scan according to the initial description by Li K, et al. [16], was used to assess COVID-19 lung involvement.

If an infection was suspected on clinical, laboratory, or imaging grounds, cultures were drawn from the suspected site. Septic shock was defined according to the Sepsis-3 criteria as a subset of sepsis in which underlying circulatory and cellular/metabolic abnormalities are profound enough to substantially increase mortality, characterized by persisting hypotension requiring vasopressors to maintain mean arterial pressure of 65 mm Hg and having a serum lactate level > 2 mmol/L (18 mg/dL) despite adequate volume resuscitation [17].

Four prognostic biomarkers were analyzed on admission and during the hospital stay, on days 7 and 14, namely:

- The neutrophil-to-lymphocyte ratio (NLR): neutrophil count/lymphocyte count
- The derived neutrophil-to-lymphocyte ratio (dNLR): neutrophil count/(white blood cell count—lymphocyte count)
- The platelet-to-lymphocyte ratio (PLR): platelet count/lymphocyte count
- The lymphocyte-to-C-reactive protein ratio (LCR): lymphocyte/C-reactive protein (mg/dL)
- The day of the recording, namely days 0, 7, and 14 were separately considered an index time (T0) for further predictions, considering only outcomes occurring strictly after the specific recording. Therefore, events occurring prior to the measurement led to the exclusion of the patient from subsequent predictive analysis (i.e., patients with septic shock occurring prior to day 14 were not included in analyzing the discriminative prowess of day 14 NLR).

### 2.3. Statistical Analysis

The statistical analysis was designed and performed by a certified biomedical statistician. The analysis was performed using the Statistical Product and Service Solution (SPSS) software, version 28.0 (SPSS Inc., Chicago, IL, USA). The Shapiro–Wilks test was used to assess distribution normality. The variables with a normal distribution were expressed as mean ± standard deviation (SD) and compared using the Student’s *t*-test. Variables with non-normal distribution were expressed as the median and interquartile range (IQR). The medians were compared with the Mann–Whitney U test. Categorical variables were analyzed using the chi-square test. The threshold for statistical significance was set at 0.05. The association between the variables of interest and two major outcomes, the development of septic shock and in-hospital mortality was tested using the Cox proportional hazards regression model. The results were expressed using the hazard ratio (HR) and 95% confidence interval (CI). Multiple multivariate scenarios were designed to avoid model overfitting and multicollinearity. Therefore, none of the hematological biomarkers (NLR, dNLR, PLR, LCR) were included within the same multivariate analysis scenario, given the close resemblance of their computational formulas (all include lymphocyte count, two include neutrophil count). The discriminative potential of NLR, dNLR, PLR, and LCR was assessed using the area under the receiver operating characteristic (AUROC) analysis.

### 2.4. Study Ethics

The current study design was discussed and approved by the host hospital Ethics Committee (46/2020, 19 November 2020). The modified 1975 Declaration of Helsinki provided the guiding ethical framework for protocol design. Informed written consent was obtained prior to inclusion from all the patients enrolled. Personal data was managed according to the European Union General Data Protection Regulation (GDPR).

## 3. Results

Our study included a consecutive series of ninety patients in the retrospective analysis, of which 50% (*n* = 45) met the septic shock criteria throughout the hospitalization. Patients who developed septic shock were significantly older and had a higher peak TSS score. However, there were no significant discrepancies between the two groups regarding the comorbidity burden (nor if assessed using the Charlson Comorbidity Index or specific underlying conditions) or conventional risk assessment tools at the time of ICU admission (SOFA or APACHE II scores) (Table 1).

The overall prevalence of culture-documented infections was 76.66% (*n* = 69). The most prevalent infection site was the respiratory system, with positive tracheal culture or sputum in 52.22% of the patients (*n* = 47), with a significantly higher proportion in patients who further developed septic shock. Bacterial or fungal respiratory infections were significantly associated with mechanical ventilation (*p* < 0.001). The most prevalent etiological agent for respiratory infection was *Acinetobacter baumannii* and *Candida albicans*, each accounting for 34.04% of infections (*n* = 16), followed by *Klebsiella pneumoniae*—29.78% (*n* = 14). The median time to the first positive culture was 13.5 days (IQR 6–19). Positive blood cultures were encountered in 20% of the patients (*n* = 18), with a significantly higher prevalence in patients who met the criteria for septic shock (31.11%, *n* = 14). The most common bacteria isolated in blood cultures was *Acinetobacter baumanii*, which was isolated in 40% of the cases (*n* = 8). The median time for meeting the criteria for septic shock was 20.5 days (IQR 16–29). Three patients developed septic shock prior to day 14 and were thus excluded from the predictive analysis regarding the biomarkers at day 14.

Regarding outcomes, patients with septic shock had a significantly higher ICU stay, had a higher rate of mechanical ventilation and need for continuous veno–venous hemodiafiltration, had a higher incidence of pulmonary thromboembolism, and, ultimately, had a significantly higher in-hospital mortality (Table 1).

**Figure 1:**
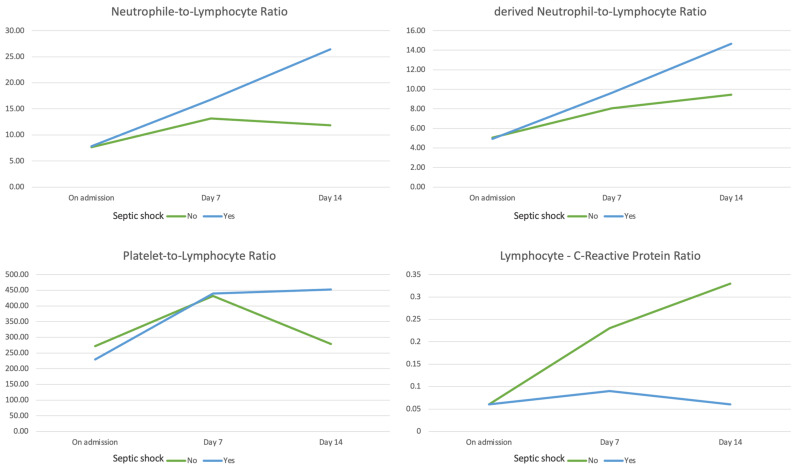
Time-dependent variation in the values of the hematological biomarkers on admission, at day 7, and at day 14.

There were no significant differences between the two groups regarding the inflammatory profile on admission, as expressed either by the C-reactive protein, procalcitonin, or by the hematological biomarkers (NLR, dNLR, PLR, and LCR). While the overall inflammatory burden increased steadily through day 7 in both groups, with no significant differences. The trends significantly diverged at day 14, with a marked increase in inflammation in patients progressing to septic shock, compared to a relative remission in patients with a favorable outcome (Figure 1).

We performed a univariate Cox-proportional hazards analysis to assess the risk for septic shock and in-hospital mortality, including the variables with significant differences between groups (Table 2). Day-14 inflammatory profiles, including CRP, NLR, dNLR, and PLR, were significant risk predictors for both progression towards septic shock and mortality.

We included the variables significantly associated with a higher risk on univariate analysis in multivariate models split into three scenarios to avoid model overfitting (as NLR, dNLR, and PLR use some of the same variables to compute). All three ratios were independent predictors for progression to septic shock and mortality (Table 3).

**Figure 2:**
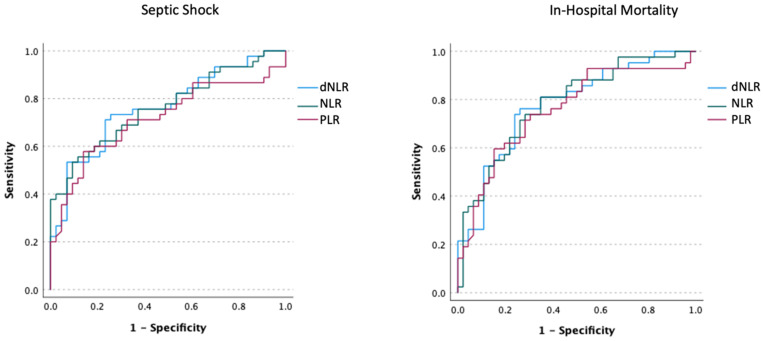
Area under the receiver-operating characteristic curve analysis for discriminating progression towards septic shock (**left**) and in-hospital mortality (**right**) for the neutrophil-to-lymphocyte ratio, derived neutrophil-to-lymphocyte ratio, and platelet-to-lymphocyte ratio.

The discriminative capabilities for predicting septic shock and mortality for NLR, dNLR, and PLR were assessed using an AUROC analysis (Figure 2). All the three ratios have shown good discrimination for septic shock, with AUROCs of 0.762, 0.764, and 0.716 for NLR (cut-off 25.33), dNLR (cut-off 9.74), and PLR (cut-off 428.49), respectively (*p* < 0.001). Similar figures were obtained for predicting mortality, with AUROCs of 0.782, 0.778, and 0.758 for NLR (cut-off 14.61), dNLR (cut-off 9.45), and PLR (cut-off 428.49), respectively (*p* < 0.001).

## 4. Discussion

Our findings suggest a high prevalence of secondary co-infections in patients hospitalized with critical COVID-19 in the ICU, which were significantly associated with mechanical ventilation. Gram-negative multi-drug resistant bacteria determined the most prevalent infections, most frequently strains of *Acinetobacter baumannii* and *Klebsiella Pneumoniae,* and frequent fungal infections, most commonly with *Candida albicans*. Secondary infections were positively correlated with the length of ICU stay. These infections tend to emerge at a later stage of hospitalization, with a median time to the first positive culture at 13.5 days. The patients who met the criteria for septic shock (50%) had significantly higher mortality (82.22% vs. 11.11%, *p* < 0.001). The median time for meeting the criteria for septic shock was 20.5 days. The cell-count ratios recorded on day 14 (NLR, dNLR, PLR) had good discriminative capabilities for predicting progression to septic shock and mortality, with AUROCs above 0.75.

Regarding our main objective, the predictive prowess of NLR, dNLR, and PLR for COVID-19 outcomes are in line with previously reported data on large cohorts of patients [11,12,13]. However, the gross data resulting from large meta-analyses should warrant a cautious interpretation, given that these ratios were studied in virtually every possible clinical design. Since the first depiction of NLR by Zahorec R in a study on oncological ICU patients, which revealed significant correlations with surgical stress, systemic inflammation and sepsis [18], the ratio has become a well-established tool in stratifying disease course, severity and overall prognosis [19]. Consideration should be made regarding the pathophysiology behind the variation in these ratios in COVID-19. Even in the early days of the pandemic, lymphopenia was regarded as a cardinal finding in the SARS-CoV-2 infection, usually serving as a quick indicator of COVID-19 while waiting for the RT-PCR results. While the exact mechanism of lymphopenia has yet to be described, there is evidence that multiple factors contribute to a drop in lymphocyte count. The lymphocytes express the angiotensin-converting enzyme 2 (ACE2) receptor, which acts as the main gateway to cell penetration for the SARS-CoV-2 virus and might ultimately lead to their lysis [20,21]. Next, there is older evidence from other clinical scenarios suggesting that multiple cytokines (IL-1, IL-6, TNF-α) are involved in generating lymphopenia by inducing apoptosis [22,23]. Thus, given that the “cytokine storm” is probably the critical component in moderate-to-severe COVID-19 [24], the rationale for lymphopenia appears relatively straightforward. Moreover, animal models have suggested that the abundance of cytokines generated in severe COVID-19 might trigger lymphoid organ atrophy and a subsequent decrease in lymphocyte count [25]. Corroborated with the potential additive effect of lactic acidosis, frequently encountered in the critically ill [20,26], there appear to be numerous ways in which lymphocyte count, the ubiquitous component in all the ratios, can drop, especially in the latter disease stages. There are also robust data reporting the predictive powers of NLR and PLR for numerous COVID-19-related outcomes in critically ill patients. Studies on similar demographics have reported good predictive value for NLR and PLR regarding the occurrence of deep vein thrombosis, acute pulmonary embolism, acute limb ischemia, need for invasive mechanical ventilation, ICU admission and mortality [27,28,29].

There appears to be a good bench-to-bedside transition regarding the role of these ratios in predicting a worse outcome. Evidence suggests that NLR’s dynamics, peak value, and values determined at specific points (at least seven days following admission) might be good predictors for mortality compared to the value on arrival [30,31]. The surge in the ratio during the hospital stay might suggest the progression towards a cytokine storm or a secondary infection, thus accurately reflecting the differences in the disease course. Our results are consistent with this hypothesis, suggesting that late spikes (day 14) in NLR, dNLR, and PLR are independent predictors for progression to septic shock and in-hospital mortality. However, evidence suggests that subsequent repetitions of these variables (i.e., day 21) bear little significance, most likely due to the patients already being on a severe disease course requiring prolonged hospital care [32].

Our results further reinforce the multiple hit progression pattern hypothesis of patients with prolonged hospitalization. The disease course is marked by an initial aggression that is determined by the severe viral infection. This requires ICU admission which is followed by the complications associated with prolonged ICU stay, most frequently represented by HAIs. The first evidence of infection is typically reported between 8 to 12 days of mechanical ventilation, and the most frequent entity is ventilator-associated pneumonia (VAP), according to a recently published large-scale systematic review [33]. According to previous reports, the most frequent VAPs were determined by multidrug-resistant strains of *Acinetobacter baumannii, Klebsiella pneumoniae,* and *Candida* spp., characterized by a marked spike in the pro-inflammatory markers and significantly increasing mortality, clearly defining a distinct clinical event [34,35,36]. Another previously published study stated that the dynamics of both NLR and dNLR were independent predictors for the need of endotracheal intubation and overall mortality in a similarly structured cohort [37]. The split in NLR trajectory throughout the hospital stay, suggesting separate disease courses, has also been described in a large, multicentric Italian study, which included 1260 critically ill COVID-19 patients. The divergence in NLR becomes increasingly evident after ten days of ICU admission, consistent with the progression to a second, distinct, disease stage [38]. Therefore, it is reasonable to suggest that there are multiple concurrent risks (i.e., HAIs, thrombotic events, neurological impairment, alteration of the nutritional status), each altering the prognosis of patients with prolonged hospitalization, according to their specific disease progression pathway. Hence, a non-linear disease course is expected, and once the disease trajectories diverge, on-admission predictors bear less significance. Although far from the realm of the current study, probably the most elegant description of this disease progression pathway is provided by Gennaro D’Amico and his colleagues in patients with liver cirrhosis and who might be consulted for further reference [39]. Following this rationale, given our focus on late decompensating events (such as septic shock, which typically occurred in the third week following admission), we believe it is reasonable to look for markers predicting these events seven days prior (i.e., at day 14), as the values on admission are far removed from the clinical state of the patient on day 21. Of course, this framework requires further validation and represents an interesting research direction, and NLR, dNLR, and PLR have promising potential. An attempt to use a competing risk model for COVID-19 patients was proposed by Zuccaro et al. in an Italian cohort, with promising results [40].

In our opinion, an exact cut-off for the ratios bears little clinical significance, as it dichotomizes a continuous variable. The futility of reporting a specific cut-off value is supported by a general lack of reproducibility, as NLR cut-offs ranged from 3.0 to 13.4, depending on the research scenario and study populations [11].

The bulk of data on the predictive value of PLR for COVID-19-associated mortality is less robust than NLR. However, two meta-analyses report that PLR can be effective in predicting disease severity [12,41]. One large-scale retrospective study has suggested that a high PLR on admission is associated with higher mortality on univariate analysis. However, on multivariate analysis, only the platelet count retained statistical significance [42]. Yet, the large scale of the study population significantly enforces the value of platelet count and PLR in assessing prognosis. The progression of the SARS-CoV-2 infection during the first waves of the pandemic was highly unpredictable, with some patients following “the calm before the storm” pathway and suffering abrupt deterioration on days 10–14. Consequently, it is reasonable to assume that on-arrival laboratory work-up might be misleading, as the dynamics of the biological variables, including peak values and repeated measurements might be more helpful. This has been hinted at by an early small-scale report from China [43], which suggested that peak PLR, rather than the values on admission, was more effective in predicting disease severity, yet no similar evidence has emerged since. To our knowledge, our study is the first to identify a predictive role of late PLR for mortality, as day 14 PLR, along with NLR and dNLR, helped determine progression to septic shock and death. Our study did not find significant discriminatory potential for LCR regarding progression to septic shock and mortality, concordant with the available literature on the topic [13].

Based on the previously discussed data, on-admission cell-count variables might be useful for predicting disease severity. Yet, once the patients become critically ill and require ICU admission, they might lose their discriminative capabilities. Therefore, dynamic close monitoring of easy-to-use, cheap, repeatable metrics might be helpful to identify an even higher vulnerability group among severe cases prone to severe secondary infections and death. Thus, using the previously discussed biomarkers might be a sensible yet not a specific surrogate to place patients on high alert and proactive treatment strategies while other time and resource-consuming methods are utilized (cultures, imaging).

Given the wide array of comorbid conditions encountered in critically ill COVID19 patients, evidence has shown that NLR might be useful in predicting the disease course of the underlying conditions, such as risk and severity of new onset acute coronary syndrome in patients with coronary artery disease [44], or to assess the activity of underlying neurological conditions [45].

Responding to the secondary objective of our research, we found a very high prevalence of HAIs, predominantly associated with mechanical ventilation. The rate of culture-documented HAIs in our study was 76.6%. On first impression, these results pinpoint a staggeringly high incidence of secondary infections. An Italian study group that analyzed data from 731 COVID-19 patients requiring hospitalization revealed a microbiologically documented infection in 9.3% of the cases. However, the study protocol included patients on the entire disease severity spectrum, and only blood cultures and lower respiratory tract cultures were considered. A subgroup analysis of 45 patients requiring ICU hospitalization within the first 48 h revealed a significantly higher co-infection rate, at 29.4% [46]. Other raw comparisons between the data are less straightforward, given that the Italian design used person-days of follow-up to calculate incidence. Another study published by Bhatt P et al. [47] on 375 patients with COVID-19 requiring supplemental oxygen documented a 34.1% prevalence of secondary infections. Navigating through their data, 47.7% were admitted to the ICU (*n* = 179), while 28.3% (*n* = 103) met the criteria for septic shock. Presuming that all the patients with criteria for septic shock were admitted to an ICU, the prevalence of septic shock among the patients in the ICU was 57.5%, a figure which closely resembles our data (*n* = 45, 50%). Similar to our results, mechanical ventilation was one of the main factors associated with HAIs. Data regarding the prevalence of secondary infections in critically ill COVID-19 patients are widely heterogeneous, as a British meta-analysis reported a 14% prevalence of bacterial co-infections [48]. In comparison, there are small-sample isolated reports of culture-documented infections in 100% of mechanically ventilated patients [49]. This raises concerns about adequate reporting and the definitions set for HAIs (screening vs. active seeking).

We acknowledge that our design has significant caveats, which warrant a cautious interpretation of our data. The sample size, monocentric, and retrospective design all diminish our findings’ significance, precluding the opportunity to perform and advanced statistical analysis. Moreover, given the retrospective analysis, a more precise disease pathway characterization was not available, as stratifying patients in different subgroups would have added substantially more information. Given the small sample size, an analysis according to the etiological agent of HAIs, the site of infection and potential mechanisms was not possible. An additional bias might reside in the inclusion of only ICU patients, thus pre-selecting for critical COVID-19 patients and canceling potential prognostic implications on the entire severity spectrum. However, given the timing of this article, we believe that prognostication of the disease course is less valuable, considering that severe forms are less common in the latter waves of the pandemic, and viral variants each have their particular natural history. Most of the cited evidence is derived from studies performed within the first year of the pandemic, when most cases were generated by the initial SARS-CoV-2 variant, alpha, and beta strains. Given the relatively milder diseases course in the subsequent variants, a pathophysiological and prognostic reconsideration might prove valuable. As a smaller proportion of patients end up critically ill, their prognostication might be substantially different, when compared to the initial cohorts. However, we believe that once a critical state occurs, the outcome depends less on the initial viral insult and more on other competing clinical events. In this light, we believe that our data on critically ill patients, with a high prevalence of patients requiring mechanical ventilation, might prove useful by extrapolation to other clinical scenarios characterized by extended ICU stay and susceptibility to HAIs.

## 5. Conclusions

The prevalence of HAIs in critically ill patients with COVID-19 is high, as almost half of the patients progress towards septic shock. The dynamics of cell-count ratios such as NLR, dNLR, and PLR might be cheap, easy-to-use, repeatable tools for discriminating progression to severe secondary infections, septic shock, and mortality.

## Figures and Tables

**Table 1:** Baseline characteristics and group comparison.

Variable	Entire Group (*n* = 90)	No Septic Shock (*n* = 45)	Septic Shock (*n* = 45)	*p*-Value
*General data*				
Age (years)	65.58 ± 11.21	62.53 ± 11.99	68.62 ± 9.56	0.009
Gender, male (*n*, %)	53 (58.88%)	25 (55.55)	28 (62.22)	0.520
Charlson Comorbidity Index	4 (4–5.4)	4 (2–6)	4 (3–7)	0.286
Obesity (*n*, %)	41 (45.55)	19 (42.22)	22 (48.88)	0.525
Diabetes mellitus (*n*, %)	39 (43.33)	17 (37.77)	22 (48.77)	0.288
Chronic pulmonary disease (*n*, %)	20 (22.22)	8 (17.77)	12 (26.66)	0.310
SOFA score at ICU admission	5 (4.8–6.1)	4 (3–6)	5 (4–9)	0.195
APACHE II score at ICU admission	15 (14.1–17.3)	14 (9.5–19)	16 (12.5–24)	0.205
Total severity score at admission	14 (11–17)	13 (10.5–16)	15 (11.5–18)	0.202
Peak total severity score	17 (13–19)	15 (12–18.5)	18 (15–19)	0.033
*Infection sites during hospital stay*				
Culture-proven infection (*n*, %)	69 (76.66)	24 (53.33)	45 (100)	<0.001
Positive tracheal culture/sputum (*n*, %)	47 (52.22)	12 (26.66)	35 (77.77)	<0.001
Positive urine culture (*n*, %)	33 (36.66)	14 (31.11)	19 (42.22)	0.274
Positive stool culture (*n*, %)	9 (10)	5 (11.11)	4 (8.88)	0.725
Clostridoides Difficile (*n*, %)	6 (6.66)	2 (4.44)	4 (8.88)	0.398
Positive wound culture—pressure ulcers (*n*, %)	4 (4.44)	0 (0)	4 (8.88)	0.041
Positive blood cultures (*n*, %)	18 (20)	4 (8.88)	14 (31.11)	0.008
*Outcomes*				
Total hospital stay (days)	24 (23.8–31.2)	23 (16–33)	25 (16.5–33.5)	1.000
Length of ICU stay (days)	11.1 (11–17.1)	8 (3–11.5)	15 (8–21)	0.001
Mechanical ventilation (*n*, %)	45 (50%)	6 (13.33)	39 (86.66)	<0.001
Continuous veno–venous hemodiafiltration (*n*, %)	16 (17.77)	2 (4.44)	14 (31.11)	<0.001
Pulmonary thromboembolism (*n*, %)	8 (8.88)	0 (0)	8 (17.77)	0.003
In-hospital mortality (*n*, %)	42 (46.66)	5 (11.11)	37 (82.22)	<0.001
*Laboratory work-up on admission*				
Hemoglobin (g/dL)	13.8 (13–13.9)	13.9 (12.25–15.05)	13.7 (11.95–15)	1.000
White blood cell count (×10^9^/L)	7.1 (6.8–9.6)	6.9 (5.37–9.72)	7.33 (5.59–11.97)	0.673
Neutrophil count (×10^9^/L)	5.8 (5.5–8.2)	5.92 (3.91–8.3)	5.77 (4.35–10.09)	0.673
Lymphocyte count (×10^9^/L)	0.8 (0.8–1.1)	0.79 (0.54–1.29)	0.82 (0.54–1.12)	1.000
Platelet count (×10^9^/L)	193 (192–238.2)	193 (150–280.5)	194 (137.5–239)	0.833
C-reactive protein (mg/dL)				
On admission	14 (12–16.9)	13 (7.36–19.15)	14.7 (5.2–22.25)	0.915
Day 7	3.88 (1.49–8.45)	2.5 (1.3–5.6)	4.3 (3–13.6)	0.033
Day 14	3.9 (1.45–8.32)	2.19 (0.81–5.86)	5.8 (2.4–14.63)	0.008
Procalcitonin (ng/mL)	0.1 (0.0–0.45)	0.1 (0.1–0.33)	0.1 (0.1–0.55)	0.522
Interleukin-6 (pg/mL)	23.1 (20–205.2)	12.52 (6.47–46.21)	58 (24–146.37)	0.004
Creatinine (mg/dL)	1.06 (0.8–1.51)	1.03 (0.8–1.32)	1.14 (0.98–1.51)	0.102
NT-proBNP (pg/mL)	506 (302.2–4 560.1)	421 (157.5–987.65)	747.5 (262–1774.25)	0.052
*Hematologic biomarkers*				
NLR				
On admission	7.65 (4.75–12.01)	7.65 (3.94–11.71)	7.82 (4.90–12.43)	1.000
Day 7	16.02 (10.49–24.93)	13.14 (6.45–20.94)	16.80 (11.08–27.60)	0.399
Day 14	20.39 (10.16–25.78)	11.84 (5.81–20.43)	26.44 (13.37–54.19)	<0.001
dNLR				
On admission	4.99 (3.05–7.38)	5.07 (2.91–7.45)	4.93 (3.22–7.48)	1.000
Day 7	8.05 (6.02–12.39)	7.54 (4.22–10.43)	9.65 (6.62–12.73)	0.092
Day 14	9.45 (4.66–16.18)	6.94 (3.31–9.72)	14.67 (7.41–19.63)	<0.001
PLR				
On admission	236.58 (149.14–353.06)	272.98 (154.47–375.55)	229.52 (153.46–323.40)	0.399
Day 7	457.14 (302.05–645.82)	432.83 (252.43–622.47)	440 (294.72–684.64)	1.000
Day 14	383.87 (246.87–539.48)	279.10 (170.14–397.91)	452.38 (277.03–681.57)	0.003
LCR				
On admission	0.06 (0.03–0.14)	0.06 (0.03–0.11)	0.06 (0.03–0.17)	0.751
Day 7	0.12 (0.06–0.44)	0.23 (0.07–0.51)	0.09 (0.04–0.30)	0.088
Day 14	0.13 (0.05–0.49)	0.33 (0.13–1.13)	0.06 (0.04–0.30)	<0.001

NLR—neutrophil-to-lymphocyte ratio; dNLR—derived neutrophil-to-lymphocyte ratio; PLR—platelet-to-lymphocyte ratio; LCR—lymphocyte-to-C-reactive protein ratio.

**Table 2:** Univariate analysis using Cox-proportional hazards model for the risk of septic shock and in-hospital mortality.

	Septic Shock			In-Hospital Mortality		
Variables	Hazard Ratio	95% Confidence Interval	*p*-Value	Hazard Ratio	95% Confidence Interval	*p*-Value
Age (years)	1.037	1.005–1.07	0.019	1.058	1.024–1.093	<0.001
Peak total severity score	0.969	0.888–1.058	0.496	0.995	0.908–1.090	0.913
Positive tracheal/sputum culture	2.162	1.052–4.441	0.036	1.935	0.962–3.892	0.064
Positive blood culture	0.845	0.413–1.726	0.643	0.989	0.484–2.023	0.976
Interleukin-6 (pg/mL)	1.000	0.999–1.001	0.708	1.000	0.999–1.001	0.911
C-reactive protein—day 14 (mg/dL)	1.006	1.002–1.010	0.005	1.007	1.002–1.011	0.002
NLR—day 14	1.029	1.015–1.042	<0.001	1.028	1.014–1.041	<0.001
dNLR—day 14	1.092	1.053–1.133	<0.001	1.087	1.049–1.128	<0.001
PLR—day 14	1.002	1.001–1.003	<0.001	1.002	1.001–1.003	<0.001
LCR—day 14	0.107	0.003–3.301	0.201	0.000	0.000–1.290	0.055

NLR—neutrophil-to-lymphocyte ratio; dNLR—derived neutrophil-to-lymphocyte ratio; PLR—platelet-to-lymphocyte ratio; LCR—lymphocyte-to-C-reactive protein ratio.

**Table 3:** Multivariate analysis using Cox-proportional hazards model for the risk of septic shock and in-hospital mortality.

	Septic Shock			In-Hospital Mortality		
Variables	Hazard Ratio	95% Confidence Interval	*p*-Value	Hazard Ratio	95% Confidence Interval	*p*-Value
*Scenario 1*						
Age	1.027	0.992–1.063	0.135	1.053	1.015–1.092	0.006
Positive tracheal/sputum culture	1.834	0.860–3.913	0.117			
C-reactive protein—day 14 (mg/dL)	1.005	1.001–1.009	0.026	1.005	1.001–1.009	0.016
NLR—day 14	1.024	1.009–1.039	0.001	1.021	1.006–1.037	0.006
*Scenario 2*						
Age	1.022	0.986–1.059	0.234	1.049	1.011–1.088	0.012
Positive tracheal/sputum culture	1.679	0.784–3.593	0.182			
C-reactive protein—day 14 (mg/dL)	1.005	1.001–1.009	0.025	1.005	1.001–1.009	0.020
dNLR—day 14	1.066	1.025–1.088	0.001	1.057	1.018–1.098	0.004
*Scenario 3*						
Age	1.027	0.994–1.062	0.111	1.052	1.016–1.091	0.005
Positive tracheal/sputum culture	1.907	0.896–4.054	0.094			
C-reactive protein—day 14 (mg/dL)	1.005	1.001–1.009	0.011	1.005	1.001–1.009	0.008
PLR—day 14	1.002	1.001–1.003	<0.001	1.002	1.001–1.003	<0.001

NLR—neutrophil-to-lymphocyte ratio; dNLR—derived neutrophil-to-lymphocyte ratio; PLR—platelet-to-lymphocyte ratio.

## Data Availability

Not applicable.
